# Exploring hospital compliance with the primary nursing care model: validating an inventory using the Delphi method

**DOI:** 10.1186/s12912-021-00712-1

**Published:** 2021-10-04

**Authors:** Antonello Cocchieri, Giorgio Magon, Manuela Cavalletti, Elena Cristofori, Maurizio Zega

**Affiliations:** 1 Fondazione Policlinico A. Gemelli IRCCS, 00168 Rome, Italy; 2 Section of Hygiene, Woman and Child Health and Public health, Università Cattolica del Sacro Cuore, Largo Francesco Vito 1, 00168 Rome, Italy; 3 European Institute of Oncology, Via Ripamonti, 435 Milan, Italy; 4 Department of Biomedicine and Prevention, University of Tor Vergata, Via Montpellier 1, 00133 Rome, Italy

**Keywords:** Content validity, Primary nursing, Checklist, Delphi method, Content validity index

## Abstract

**Background:**

The primary nursing care model is considered a personalized model of care delivery based on care continuity and on the relationship between the nurse and patient. Primary nursing checklists are not often mentioned in the literature; however, they represent a valid instrument to develop, implement, and evaluate primary nursing. The aim of this study was to create a structured checklist to explore hospital compliance in primary nursing.

**Methods:**

The Delphi method was used to develop and validate a checklist. The preliminary version was created and sent to three experts for their opinions. Their comments were ultimately used in the first version, which included four components with 19 items regarding primary nursing characteristics. A two-round Delphi process was used to generate consensus items. The Delphi panel consisted of six experts working in primary nursing contexts and/or teaching or studying primary nursing. Data were collected using a structured questionnaire from July 2020 to January 2021. These experts were asked to rate each element for relevance using a 4-point Likert-type scale. Furthermore, the consensus among the panel of experts was set at ≥78%, with selected items being voted “quite relevant” and “highly relevant”. Content validity index (I-CVI) and modified kappa statistic were also calculated. Following expert evaluation, the first version of the checklist was modified, and the new version, constituting 17 items, was sent to the same experts.

**Results:**

The first version of the checklist demonstrated a main relevance score of 3.34 (SD = 0.83; range = 1.3–4; mean I-CVI = 0.84; range: 0.83–1), but three items did not receive an adequate I-CVI score, that is, lower than 0.78. After the second round, the I-CVIs improved. The main score of relevance was 3.61 (SD: 0.35; range = 2.83–4; mean = I-CVI: 0.93). The S-CVI/UA was 0.58, and the S-CVI/Ave was 0.93.

**Conclusion:**

Measuring primary nursing compliance should be implemented to provide continuous feedback to nurses. Moreover, utilizing valid checklists could permit comparing different results from others’ research. Future research should be conducted to compare the results from the checklist with nursing outcomes.

**Supplementary Information:**

The online version contains supplementary material available at 10.1186/s12912-021-00712-1.

## Background

Nursing care delivery models (NCDMs) are defined as specific forms of care delivery in response to demographic, organisational, and healthcare environment changes [1]. In the literature, NCDMs are described as independent or collaborative approaches by nurses to provide direct care to a group of patients [2]. Over time, traditional NCDMs have evolved based on economics from the 1930s to the present and include approaches, such as total patient care, functional care, team nursing care, and the primary nursing care model (PN) [3]. They can differ from each other in terms of philosophy, the conceptual model utilized, work allocation, nursing staff, patient allocation, skill mix, and costs, with the common goal of improving the quality of nursing care [4].

PN is considered a personalized model of care delivery based on care continuity and on the relationship between nurses and patients. PN can be described as a delivery system comprised of four organisational elements, which differentiate PN from other systems, such as functional nursing or team nursing [3]. These elements are summarised in four principal points: a) responsibility for relationship and decision-making, b) work allocation and patient assignments, c) communication among staff members, and d) management and leadership philosophy [5]. In PN, the primary nurse is responsible for several patients 24 h per day, 7 days a week, including during off-duty hours. The primary nurse is a registered nurse responsible for planning, providing, and evaluating the care of patients throughout their stay in hospitals [6, 7].

Evaluating the impact of NCDMs is a fundamental part of clinical practice improvement, and the evaluation of hospital compliance with related principles and organisational rules is the first base element [8]. Even though the term “compliance” has many definitions [9], we approach it from an evaluative perspective [10]. In particular, we consider compliance as behaviour conforming to rules or specific standards, such as PN principles.

Studies that have evaluated the impact of PN have used both quantitative and qualitative methods [5, 11]. Studies related to PN have utilized different outcomes for determining their efficacy, such as the effect on care quality [12], positive patient experience [12, 13], costs [14], or other nursing outcomes [11, 15]. However, various difficulties have been discovered in analysing these studies because PN differs in terms of the manner and time in which it is implemented [12, 14]. These results are often incomparable because certain elements of PN remain unclear or unspecified [14, 15].

To facilitate the development, implementation, and evaluation of an NCDM, focusing on the role of nursing [16] in terms of development strategies [17] and system evaluation control is crucial [18].

One way to evaluate the implementation of processes is by using checklists [19], which are used in many contexts to aid decision-making [20]. A checklist is a structured inventory that outlines the criteria of consideration for a specific process [19, 20]. They are used to remember all relevant criteria that should be considered in one particular aspect. The introduction of checklists in healthcare has had a positive effect in terms of improving the quality of care and reducing negative outcomes [21]. The literature recommends using standardised methodologies for checklist design [22].

PN checklists are lacking in the literature. Because checklists are a valid instrument to develop, implement, and evaluate PN, the aim of this study is to create a structured checklist to explore hospital compliance with the PN.

## Methods

This is a methodological study employing the Delphi method [23] to develop and validate a checklist to evaluate compliance in a specific hospital setting with PN elements based on the main principles described by Manthey [7].

### Development of a checklist to evaluate compliance in a hospital setting with PN elements

The preliminary version was created by two researchers in nursing science to evaluate hospital adherence to PN principles. The authors developed a preliminary version based on the discussion about the main areas constituting PN: (a) the decision-making process (the primary nurse should be responsible for patient-centered decision-making); (b) work allocation and/or patient assignment (strategies for daily care and for assigning the nurse to do this care); (c) communication (direct interpersonal communication with other staff about the patient); and (d) accountability (being accountable for the quality of care provided for a fixed caseload of patients 24 h a day, 7 days a week) [5]. The researcher included items referring to these areas.

This preliminary version was sent to a professor in nursing science and two nursing service managers to give their opinion about the items and suggest other relevant aspects to complete the inventory tool. The nursing service managers were experts in applying PN, as they have developed and implemented PN since 2010 in two Italian academic hospitals. Comments were collected using a checklist with the possibility of entering any comment in free text per each item. The checklist was sent via email from February 2020 to March 2020 after obtaining the experts’ written consent.

At the end of this revision, the structure of the first version of the checklist included four areas: decision-making process (five items), work allocation and patient assignment (five items), communication (five items), and accountability (four items), with a total of 19 items regarding primary nursing characteristics (Additional file 1). Moreover, the first version of the checklist was created with the option of entering any comment in free text.

### Validation of the checklist

In the first round of evaluation, the first version of the checklist was sent to six nurse experts in PN, who belonged to a panel of experts [23]. Four of them had contributed to the development of a PN model in hospital settings. The selection criteria for the expert panel included a minimum of 2 years of experience working with PN and/or teaching or studying this nursing care model. Four females and two males constituted the panel. They had a mean age of 38 years (± 9.8; range: 30–50) and had been employed as registered nurses for a mean of 14.6 years (± 11.78; range: 4–30). Their professional roles ranged as follows: nursing clinical care (*n* = 2), nursing management (*n* = 3), and one researcher in nursing science. Their academic titles were Bachelor of Nursing Science (*n* = 2), Master of Science in Nursing (*n* = 3), and Doctor of Philosophy in Nursing (*n* = 1). These experts were asked to rate each element for relevance using a 4-point Likert-type scale (1 = not relevant, 2 = somewhat relevant, 3 = quite relevant, and 4 = highly relevant) according to Lynn, Polit, and Beck [24]. They were also asked to provide comments at the end of the checklist to add any other useful element. Data were collected using a structured questionnaire from July 2020 to November 2020 after obtaining the experts’ written consent to participate.

The responses were used to calculate the content validity at the item and scale levels. The content validity of individual items (I-CVI) was calculated as the number of experts giving a rating of 3 or 4 (dichotomizing the scale into relevant and not relevant) divided by the total number of experts [23, 24]. As recommended by Lynn, I-CVI should not be lower than 0.78 [23].

Also, a multi-rater Kappa statistic was used to adjust I-CVI for chance agreement [25, 26]. The Kappa is a consensus index used to test inter-rater agreement among experts who rate dichotomous categories of data and indicates beyond chance the relevance of the item [25–27]. A modified Kappa (K*) [25, 26] value was computed using the following formula: K* = (I-CVI-P_C_) / (1-P_C_). P_C_ is the probability of chance agreement and was calculated for each item with the following formula: P_C_ = [N! /A! (N-A)!]*0.5^N^ [25], where N = number of experts, and A = number of experts who agree the item is relevant. K* values above 0.74 were considered excellent, between 0.60 to 0.74 good, and 0.40 to 0.59 fair. Values should not be lower than 0.60 [25].

Finally, the content validity at the scale level was measured through the scale-level content validity index, the universal agreement calculation method (S-CVI/UA), and the average of the I-CVIs for all items of the scale (S-CVI/Ave). The S-CVI/UA was calculated as the proportion of total agreement (from I-CVI scores) by all the experts, and the S-CVI/Ave as the average of all I-CVIs.

### Second version of checklist

After the first round, the first version of the checklist was modified, and the new version (second version—see Additional file 2), constituting 17 items, was sent to the same experts as recommended in [23, 24]. The response rates were calculated in the same manner as in the first round. Data were collected from December 2020 to January 2021.

### Final version of the checklist

The final version of the checklist was sent to the same experts for final approval, and they were asked to add any comments they may have.

## Results

### Development of the checklist to evaluate the compliance of hospital setting to PN elements

During the development of the preliminary version of the checklist, three experts helped improve the items. They contributed by adding aspects regarding the nurses’ competence evaluations and their patients’ assignment modalities. In particular, they expressed the necessity to monitor and evaluate the compliance of wards to PN to evaluate the impact on nursing outcomes. Some statements are reported below.

“This inventory could be a way to improve nursing quality. This could be considered a good system to control compliance. This inventory could be used as tracer methodology.”

“Evaluating helps to learn how aspects could be improved and it’s a good technique to learn.”

“It’s necessary to add an item on nurses’ competences. The objective evaluation of PN improves the adherence of nurses equips.”

These comments were used to improve and develop the first version of the checklist.

### Validation of the checklist

The first version of the checklist, after the first round, showed a main score of relevance of 3.34 (DS 0.83¸ range = 1.3–4). The checklist showed an excellent I-CVI for 15 items out of a total of 19 (mean I-CVI = 0.83; range: 0.83–1), but three items did not receive an adequate I-CVI score, that is, lower than 0.78 according to Lynn (1986). Items receiving lower CVI ratings were consistent with items with lower Kappa coefficients (Table 1). A test score of an I-CVI of ≥0.78 was equivalent to a probability of a P_C_ < 0.09, indicating an excellent level of expert agreement concerning the test’s relevance (Table 1).

**Table 1:** I-CVI ratings and Kappa Score on a 19-item checklist by six experts: Items 3 and 4 on a 4-point Relevance Scale

N	Item	Number in agreement	I-CVI	Interpretation	P_C_	K*	Interpretation
1	The primary nurse has a direct interpersonal communication with the patient (the patient knows his/her primary nurse)	6	1	Relevant	0.016	1	Excellent
2	The primary nurse has a direct interpersonal communication with the caregiver (the caregiver/family knows the patient’s primary nurse)	6	1	Relevant	0.016	1	Excellent
3	The primary nurse is able to introduce him/herself to the patient	6	1	Relevant	0.016	1	Excellent
4	The primary nurse (or nurse coordinator) delegates the care of the patients to the associated care unit in case of any absences	6	1	Relevant	0.016	1	Excellent
5	There is evidence of face-to-face interactions between nurses and patients	5	0.83	Relevant	0.094	0.81	Excellent
6	There is evidence about who is the primary nurse of each inpatient	1	0.16	Eliminated	0.094	0.07	Unacceptable
7	There is evidence of primary nurse assignment by the nurse coordinator through weekly planning	4	0.66	Modified	0.234	0.57	Fair
8	The competence of the primary nurses is evaluated by the nurse coordinator	6	1	Relevant	0.016	1	Excellent
9	There is evidence of an evaluation of the nurses’ competencies by a valid and reliable instrument	2	0.33	Eliminated	0.234	0.12	Unacceptable
10	There is no evidence of practicing other nursing care models	5	0.83	Relevant	0.094	0.81	Excellent
11	The primary nurse has direct interpersonal communication with the physician concerning the clinical status of the patient	5	0.83	Relevant	0.094	0.81	Excellent
12	The primary nurse has direct interpersonal communication with other members of the staff concerning the patient and his/her clinical status	5	0.83	Relevant	0.094	0.81	Excellent
13	The primary nurse is accountable for the quality of the care provided to the patient	6	1	Relevant	0.016	1	Excellent
14	The patient is well-informed by the primary nurse about the details of his/her care plan	5	0.83	Relevant	0.094	0.81	Excellent
15	Daily focuses are organised among nurses on the main needs of patients	5	0.83	Relevant	0.094	0.81	Excellent
16	The name of the primary nurse is reported in the clinical documentation	6	1	Relevant	0.016	1	Excellent
17	The nursing plan of care is reported in the clinical documentation	6	1	Relevant	0.016	1	Excellent
18	There is evidence of the patient’s evaluation within 24 h of admission	6	1	Relevant	0.016	1	Excellent
19	There is evidence of the patient’s evaluation after 7 days of hospitalisation	5	0.83	Relevant	0.094	0.81	Excellent

*I-CVI* Item-level content validity index, *P*_*C*_ probability of a chance occurrence, K* Modified Kappa index

Two items (Item 6, “There is evidence about who is the primary nurse of each impatient” and Item 9, “There is evidence of an evaluation of the nurses’ competencies by a valid and reliable instrument”) were removed because their I-CVI values were 0.16 and 0.33, respectively, and because they referred to topics that were in other items. Item 6 was removed because it was already included in Item 16, “The name of the primary nurse is reported in the clinical documentation.” Item 9 was removed because it was already included in Item 8, “The competence of the primary nurse is evaluated by the nurse coordinator”. Item 7 had an I-CVI of 0.66 and was modified as requested by experts from “There is evidence of primary nurse assignment by the nurse coordinator through weekly planning” to “There is evidence of primary nurse assignment by the nurse coordinator through planning.” The S-CVI/UA was 0.47, and S-CVI/Ave was 0.83.

### Second version and final approval of the checklist

After the second round, the I-CVIs improved. The main score of relevance was 3.61 (DS: 0.35; range = 2.83–4). The checklist showed an excellent I-CVI for all items, which ranged from 0.83 to 1 (mean I-CVI: 0.93). Also in this version, items receiving lower CVI ratings were consistent with items with lower Kappa coefficients (Table 2).

**Table 2:** I-CVI ratings and Kappa Score on a 17-item checklist by six experts: Items 3 and 4 on a 4-point Relevance Scale

N	Item	Number in agreement	I-CVI	Interpretation	P_C_	K*	Interpretation
1	The primary nurse has a direct interpersonal communication with the patient (the patient knows his/her primary nurse)	6	1	Relevant	0.016	1	Excellent
2	The primary nurse has a direct interpersonal communication with the caregiver (the caregiver/family knows the patient’s primary nurse)	6	1	Relevant	0.016	1	Excellent
3	The primary nurse is able to introduce him/herself to the patient	6	1	Relevant	0.016	1	Excellent
4	The primary nurse (or nurse coordinator) delegates the care of the patients to the associated care unit in case of any absences	6	1	Relevant	0.016	1	Excellent
5	There is evidence of face-to-face interactions between nurses and patients	5	0.83	Relevant	0.094	0.81	Excellent
6	There is evidence of primary nurse assignment by the nurse coordinator through a planning	5	0.83	Relevant	0.094	0.81	Excellent
7	The competence of the primary nurses is evaluated by the nurse coordinator	6	1	Relevant	0.016	1	Excellent
8	There is no evidence of practicing other nursing care models	5	0.83	Relevant	0.094	0.81	Excellent
9	The primary nurse has direct interpersonal communication with the physician concerning the clinical status of the patient	5	0.83	Relevant	0.094	0.81	Excellent
10	The primary nurse has direct interpersonal communication with other members of the staff concerning the patient and his/her clinical status	5	0.83	Relevant	0.094	0.81	Excellent
11	The primary nurse is accountable for the quality of the care provided to the patient	6	1	Relevant	0.016	1	Excellent
12	The patient is well-informed by the primary nurse about the details of his/her care plan	5	0.83	Relevant	0.094	0.81	Excellent
13	Daily focuses are organised among nurses on the main needs of patients	6	1	Relevant	0.016	1	Excellent
14	The name of the primary nurse is reported in the clinical documentation	6	1	Relevant	0.016	1	Excellent
15	The nursing plan of care is reported in the clinical documentation	6	1	Relevant	0.016	1	Excellent
16	There is evidence of the patient’s evaluation within 24 h of admission	6	1	Relevant	0.016	1	Excellent
17	There is evidence of the patient’s evaluation after 7 days of hospitalisation	5	0.83	Relevant	0.094	0.81	Excellent

*I-CVI* Item-level content validity index, *P*_*C*_ probability of a chance occurrence, K* Modified Kappa index

Consensus items generated from the first and second rounds are shown in Table 3.

**Table 3:** Consensus items generated from Rounds 1 and 2

	Numbers in agreement		I-CVI		K*	
	First version	Second version	First version	Second version	First version	Second version
1	6	6	1	1	1	1
2	6	6	1	1	1	1
3	6	6	1	1	1	1
4	6	6	1	1	1	1
5	5	5	0.83	0.83	0.81	0.81
6	1	R	0.16	R	0.07	R
7	4	M (5)	0.66	0.83	0.57	0.81
8	6	6	1	1	1	1
9	2	R	0.33	R	0.12	R
10	5	5	0.83	0.83	0.81	0.81
11	5	5	0.83	0.83	0.81	0.81
12	5	5	0.83	0.83	0.81	0.81
13	6	6	1	1	1	1
14	5	5	0.83	0.83	0.81	0.81
15	5	6	0.83	1	0.81	1
16	6	6	1	1	1	1
17	6	6	1	1	1	1
18	6	6	1	1	1	1
19	5	5	0.83	0.83	0.81	0.81

*I-CVI* Item-level content validity index, K* Modified Kappa index, *R* removed, *M* modified

The S-CVI/UA was 0.58, and the S-CVI/Ave was 0.93 (Table 4).

**Table 4:** SCVI/Ave and S-CVI/UA generated from Rounds 1 and 2

SCVI/Ave		S-CVI/UA	
First version	Second version	First version	Second version
0.83	0.93	0.47	0.58

In the final approval, no additional comments were expressed.

## Discussion

This study presents quantity indices for content validity and a new checklist that assesses areas of PN relating to the principal four elements of the associated framework [7]. We previously did not find any research involving the development of inventory to monitor hospital compliance with the PN. Various studies have identified specific characteristics in defining the development process of PN [12–14], but evidence about a valid tool was not found.

Our results demonstrate an improvement in the I-CVI index at each round. They showed excellent agreement among experts in terms of item scoring after the second round. In the literature, the I-CVI index should be 1.00 if the experts are fewer than five. An I-CVI of at least 0.78 should be seen as adequate with six or more experts. I-CVI rates were considered in changing or revising any items in our draft.

Both S-CVI were calculated [24]. S-CVI/UA showed an improvement from 0.47 to 0.58. When calculating S-CVI/UA, the universal agreement of experts was considered, and if the number of experts increased, S-CVI/UA would be low [24]. For this reason, we calculated S-CVI/Ave as recommended in [24]. Our results were higher than .90, which showed an excellent average proportion of relevant rates (3 and 4) evaluated by experts.

The first important element of PN is responsibility for relationships and decision-making [5, 7]. Experts agreed with the importance of developing direct interpersonal communication with the patient (Item 1; I-CVI =1) and with the caregiver (Item 2; I-CVI = 1). This reflects the actual practice of PN, which incorporates the principles of relationship-based care into daily practice, identifying the primary nurse as responsible for the relationship [7]. In this role, the primary nurse, with the patient and caregivers, identifies the needs of the patients and creates a personalized nursing care plan. Because the PN supports individual care, the experts agreed with the importance of developing a personalized nursing care plan and reported it in clinical documentation (Item 15; I-CVI =1).

Communication among staff members is also an important element of PN. The quality of care depends on the information flow between the patient and all members of the healthcare team. Experts agreed with the necessity to increase communication between the people involved in patient care (Items 9 and 10; I-CVI 0.83), as well as more accurate information about patients (Item 12; I-CVI =0.83). PN is about creating healthy partnerships between nurses and other clinicians to best serve patients and their families [7].

Furthermore, managers should support PN organisation. They are committed to creating opportunities for professional growth. They should guarantee that no other nursing care models are performed (Item 8; I-CVI = 0.83). They are also committed to the development of clinical staff and the evaluation of nurses’ competence to improve quality of care [5, 13].

Finally, work allocation and patient assignments are required in PN care. The nursing care plan must be followed by the other nurses—associate nurses—and the primary nurse can delegate the patient care to the associated care unit in case of absences [7]; the experts agreed with this (Item 4; I-CVI =1). Experts’ discussion about “the evidence of primary nurse assignment by nurse coordinator through weekly planning” (first version of Item 7; I-CVI =0.66) highlights the importance of nurse assignment to patients but not that of frequency of how it is planned. However, they recommended providing the team responsibility assignment with the “evidence of the primary nurse’s name in the clinical documentation” (Item 16; I-CVI =1).

This checklist has potential applications in both the research setting and clinical practice. Researchers can use this checklist to describe more accurately the PN characteristics and compare results from different research using the same instrument. In clinical practice, the checklist provides a unique instrument to support hospital team members during the PN implementation and evaluation phase. In fact, the discordance and discussions surrounding the manner in which it is implemented [12, 15] suggest the need for a valid guide. Monitoring and evaluating PN are both strategies that help nurses know when a planned project is not working. An effective PN compliance management system could allow organisations to direct quality improvement actions where the PN is more critical.

This study has some limitations. Although the selection of a panel of experts was carefully made to ensure high-level competency concerning the PN, the preliminary version of the checklist was created by two researchers in nursing science, and they could not identify the entire domain of PN. Moreover, our experts worked in Italian acute hospital settings and specific contexts were not considered. Finally, this checklist should be tested in clinical practice.

## Conclusion

In summary, in this study, we developed a valid checklist for implementing and monitoring PN compliance in hospital settings. Ongoing monitoring is built into normal, recurring nursing activities for quality improvement. Measuring PN compliance should be applied to provide continuous feedback to nurses. Using this tool could create specific moments of learning and education. Nursing programmes and courses could be considered to maintain adequate nurse competences in PN. Moreover, utilising a valid checklist could permit the comparison of different results by other researchers, which is not currently being done. Future research should be conducted to compare the results from the checklist with nursing outcomes.

## Supplementary Information

**Additional file 1.**

**Additional file 2.**

## Data Availability

All data generated or analysed during this study are included in the published article.

## Abbreviations

NCDM: Nursing care delivery models
PN: Primary nursing care model
I-CVIs: Content validity index
S-CVI/UA: Scale-level content validity index, universal agreement calculation method
S-CVI/Ave: Scale-level content validity index, averaging calculation method
P_C_: Probability of a chance occurrence
K*: Modified Kappa index
