# Supplementary material for: Exploring hospital compliance with the primary nursing care model: validating an inventory using the Delphi method
Source: BMC Nurs. 2021 Oct 4;20:188. doi: 10.1186/s12912-021-00712-1 (PMC8491371; doi:10.1186/s12912-021-00712-1)
Supplement: Supplementary file 2 — Additional file 2. [file 12912_2021_712_MOESM2_ESM.docx]

**Additional file 2**

|  |  | Not relevant | Somewhat relevant | Quite relevant | Highly relevant |
| --- | --- | --- | --- | --- | --- |
| **Decision-making process** | | | | | |
| 1 | The primary nurse has a direct interpersonal communication with the patient (the patient knows his/her primary nurse) | 1 | 2 | 3 | 4 |
| 2 | The primary nurse has a direct interpersonal communication with the caregiver (the caregiver/family knows the patient’s primary nurse) | 1 | 2 | 3 | 4 |
| 3 | The primary nurse is able to introduce him/herself to the patient | 1 | 2 | 3 | 4 |
| 4 | The primary nurse (or nurse coordinator) delegates the care of the patients to the associated care unit in case of any absences | 1 | 2 | 3 | 4 |
| 5 | There is evidence of face-to-face interactions between nurses and patients | 1 | 2 | 3 | 4 |
|  | **Work allocation and patient assignment** |  |  |  |  |
| 6 | There is evidence of primary nurse assignment by the nurse coordinator through a planning | 1 | 2 | 3 | 4 |
| 7 | The competence of the primary nurses is evaluated by the nurse coordinator | 1 | 2 | 3 | 4 |
| 8 | There is no evidence of practicing other nursing care models | 1 | 2 | 3 | 4 |
|  | **Communication and management of the unit** |  |  |  |  |
| 9 | The primary nurse has a direct interpersonal communication with the physician concerning the clinical status of the patient | 1 | 2 | 3 | 4 |
| 10 | The primary nurse has a direct interpersonal communication with other members of the staff concerning the patient and his/her clinical status | 1 | 2 | 3 | 4 |
| 11 | The primary nurse is accountable for the quality of the care provided to the patient | 1 | 2 | 3 | 4 |
| 12 | The patient is well-informed by the primary nurse about the details of his/her care plan | 1 | 2 | 3 | 4 |
| 13 | Daily focuses are organised among nurses on the main needs of patients | 1 | 2 | 3 | 4 |
|  | **Management of the unit or environment of care** |  |  |  |  |
| 14 | The name of the primary nurse is reported in the clinical documentation | 1 | 2 | 3 | 4 |
| 15 | The nursing plan of care is reported in the clinical documentation | 1 | 2 | 3 | 4 |
| 16 | There is evidence of the patient’s evaluation within 24 hours of admission | 1 | 2 | 3 | 4 |
| 17 | There is evidence of the patient’s evaluation after 7 days of hospitalisation | 1 | 2 | 3 | 4 |
